# Treatment of Gangrene Infection

**Published:** 1918-10

**Authors:** 


***Note on the Treatment of Gangrene Infection of War Wounds.*** L. Ombredanne, *Bulletin et Mémoires de la Société de Chirurgie de Paris.* Vol. XLI. N° 4.

After a very large debridement and removal of projectiles and foreign bodies, and after making parallel incisions in the whole area of crepitation, the author washes out the wound with ether, plugging it with gauze filled with ether; gauze with ether is packed under the strips of skin, ether compresses are applied on the teguments, and the whole thing is covered up with a rubber cloth.

The dressing is changed completely, morning and night, for three or four days.

The results have been wonderful, and infection has been stopped in every case within three to six days

